# Integrated analysis for treatment scheme of sodium–glucose cotransporter 2 inhibitors in patients with diabetic kidney disease: a real-world study

**DOI:** 10.1038/s41598-023-33211-1

**Published:** 2023-04-12

**Authors:** Li Fang, Guangpu Li, Jingjing Ren, Jiayu Duan, Jiancheng Dong, Zhangsuo Liu

**Affiliations:** 1 Department of Integrated Traditional and Western Nephrology, The First Affiliated Hospital of Zhengzhou University, Zhengzhou, China; 2 Research Institute of Nephrology, Zhengzhou University, Zhengzhou, China; 3 Henan Province Research Center for Kidney Disease, Zhengzhou, China; 4 Key Laboratory of Precision Diagnosis and Treatment for Chronic Kidney Disease in Henan Province, Zhengzhou, China; 5 Clinical Research Center of Big-Data, The First Affiliated Hospital of Zhengzhou University, Zhengzhou, China

**Keywords:** Endocrinology, Health care, Medical research, Nephrology

## Abstract

Sodium–glucose cotransporter 2 inhibitors (SGLT2i) are recommended for type 2 diabetes mellitus patients with impaired renal function, but the actual situation of SGLT2i using is unclear. Therefore, in this real-world study, we analyzed the treatment scheme and clinical characteristics of SGLT2i in patients with diabetic kidney disease (DKD). We included DKD patients hospitalized in the First Affiliated Hospital of Zhengzhou University from October 2017 to March 2020. The Apriori algorithm of association rules was used to analysis treatment scheme prescribing SGLT2i and other different combinations of hypoglycemic drugs. SGLT2i was used in 781 (12.3%) of 6336 DKD patients, both number and proportion of patients using SGLT2i increased from 2017 to 2020 (1.9% to 33%). Nighty-eight percent of all DKD patients using SGLT2i were combined with other glucose-lowering agents, and insulin, metformin and alpha-glucosidase inhibitors are most commonly used in combination with hypoglycemic drugs. Multivariate analysis showed that compared with non-SGLT2i group, patients using SGLT2i were associated with younger age, higher BMI, higher HbA1c, preserved kidney function, dyslipidemia and combined with ACEI/ARB and statins. In this real-world study, use of SGLT2i in DKD patients is still low. Most patients performed younger age and in the early stages of chronic kidney disease with poor glycemic control. Clinical inertia should be overcome to fully exert the cardiorenal protective effects of SGLT2 inhibitors, with attention to rational drug use.

## Introduction

Diabetic kidney disease (DKD) has been one of the most serious complications of diabetes and the leading cause of end-stage renal disease (ESRD)^1,2^. Comparing with Caucasians, Asian patients with type 2 diabetes mellitus (T2DM) performed higher prevalence of DKD and faster deterioration of renal function^3^. Therefore, it’s an urgent to find effective therapeutic interventions since the occurrence and development of DKD contributed to increased morbidity, mortality and seriously affect the quality of life^4^.

Previous large-sample randomized clinical trials, including CANVAS, DECLARE-TIMI 58 and EMPA-REG, evaluated the efficacy and efficiency of sodium–glucose cotransporter 2 inhibitors (SGLT2i) and provided growing evidences indicating the beneficial effects on renal function in patients with T2DM^5–7^. With the publication of the CRENDENCE study results, significant cardiorenal benefits were shown in patients with estimate glomerular filtration rate (eGFR) as low as 30 ml/min/1.73 m^2^, and similar results were seen in the DAPA-CKD trial^8,9^. SGLT2 inhibitors now are recommended as first-line agents for T2DM with impaired renal function^10^.

However, randomized controlled trials (RCTs) are designed using strict screening criteria for participants and predefined protocols. Their results are pure and restricted, which could not fully reflect the whole picture of usage and efficacy of treatment in clinical practice. For real-world studies, where inclusion and exclusion criteria are relatively broad and treatment measures are selected non-randomly based on the patients' condition and wishes, the results are more relevant to the reality of the situation. At present, there have been some real-world studies of SGLT2i in western countries^11–13^, but there may be some variation in drug use among different ethnic groups due to the characteristics of the populations and medical consultations. Therefore, we selected a representative hospital in central China to explore the use and clinical characteristics of SGLT2i in DKD patients and to analyze the combination of SGLT2i with other hypoglycemic drugs, so as to provide reference for clinical medication in Asian patients.

## Methods

### Study population and design

Hospitalized patients who were diagnosed with diabetic kidney disease in the First Affiliated Hospital of Zhengzhou University from October 2017 to March 2020 were included in our study. Among these patients, those who received SGLT2 inhibitors including canagliflozin, dapagliflozin and empagliflozin during this period were selected, and patients receiving non-SGLT2i were set as the control group. Patients were required to be new users with no history of SGLT2i exposure in the past year. Patients were excluded if they were younger than 18 years, had a diagnosed with type 1 diabetes, gestational diabetes, cancer, acute ketoacidosis or dialysis.

### Data collection

Data was obtained from electronic medical records system (EMR), including patients’ demographic characteristics, results of laboratory tests and usage of medication. For multiple admissions, the data from the patient’s last admission were used. We described the trend of SGLT2i use over time from October 2017 to March 2020 by quarter and analyzed the factors influencing SGLT2 inhibitor use.eGFR was calculated by the Chronic Kidney Disease-Epidemiology Collaboration (CKD-EPI) equation based on age, gender, race and serum creatinine. There were 6 stages of CKD according to eGFR, in order of G1, ≥ 90; G2, 60–89; G3a, 45–59; G3b, 30–44; G4, 15–29 and G5, < 15 ml/min/1.73 m^2^. Urinary albumin excretion was classified into three classes A1-A3, < 30, 30–300 and > 300 mg/g respectively. Furthermore, we defined categorical variables: Anemia was defined as hemoglobin < 120 g/L in males and < 110 g/L in females and hyperuricemia was defined as serum uric acid > 420 μmol/L in male patients and > 360 μmol/L in female patients.

In addition, combinations with SGLT2i were analyzed, including five oral and two injectable hypoglycemic drugs: metformin, alpha-glucosidase inhibitors (AGIs), sulfonylurea (SU), dipeptidyl peptidase-4 inhibitors (DPP-4i), glinide, glucagon-like peptide-1 receptor agonists (GLP-1RA) and insulin. According to renal function, patients were divided into three groups: eGFR ≥ 60, eGFR 30–59 and eGFR < 30 ml/min/1.73 m^2^ to analyze the drug combination patterns.

This study was approved by the Ethics Committee of the First Affiliated Hospital of Zhengzhou University and waived the need for informed consent due to the EMR data was authorized for research purpose by the National Health Commission of the People’s Republic of China (http://www.nhfpc.gov.cn/). All methods were performed in accordance with relevant guidelines and regulations.

### Statistical analysis

Data was expressed as means with standard deviations (SD), medians with interquartile range or frequency with percentage, as appropriate. Intergroup comparisons were performed by Pearson Chi-square test, Student’s t-test, Mann–Whitney U-test and Wilcoxon test for categorical and continuous variables, as appropriate. Univariate and multivariable logistic regression model was fitted to assess factors associated with using of SGLT2i. Results were reported as odds ratio (OR) with 95% confidence intervals (CI). The variance inflation factor was used to check for multicollinearity. Sensitivity analyses was performed in DKD patients with eGFR ≥ 45 ml/min/1.73 m^2^ under conditions eligible for SGLT2 inhibitor use. In multivariable models, missing data were handled by multiple imputation models using the mice package. The Apriori algorithm of association rules was used to analyze the combination of SGLT2 inhibitors. The strength of association rules is determined by support (S) and confidence (C). S (X → Y) = P (X → Y) represents the probability of X and Y occurring at the same time. In the sample data, it represents the frequency of X and Y occurring at the same time, X and Y represent other hypoglycemic drugs. C (X → Y) = P (X/Y) = S (X → Y)/S (X), is the conditional probability of Y occurring under the condition that X occurs. In the sample data, it represents the frequency of Y in the number of cases that X occurs. In this study, the minimum support and confidence levels were set to 10% and 60%, respectively, and the minimum item set was 2. The network diagram was used to represent the association rules for the combination of other hypoglycemic drugs. The results of the network diagrams are presented as connecting lines, with thicker lines indicating a higher frequency and closer association. In addition, the sunburst chart was used to show the combination patterns of patients with different renal function. The center of sunburst chart shows the patients who were using SGLT2i, the first ring shows the proportion of patients who were initiated with one other hypoglycemic drug, and the second ring shows the proportion of patients who were initiated with the second other hypoglycemic drug. A *P*-value < 0.05 was considered statistically significant. All the analyses were performed on R version 4.1.1 and SPSS Modeler 18.0.

## Results

### Baseline characteristics of the overall study population

**Figure 1:**
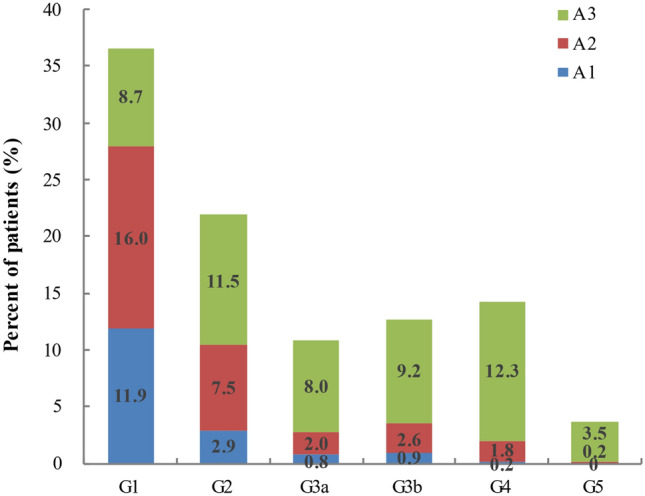
Proportion of patients with DKD in each eGFR and albuminuria category. A1–A3 denote albuminuria category. Albuminuria categories: A1, < 30 mg/g; A2, 30–300 mg/g; A3, > 300 mg/g. G1–G5 denote GFR categories: G1, ≥ 90 ml/min/1.73 m^2^; G2, 60–89 ml/min/1.73 m^2^; G3a, 45–59 ml/min/1.73 m^2^; G3b, 30–44 ml/min/1.73 m^2^; G4, 15–29 ml/min/1.73 m^2^; G5, < 15 ml/min/1.73 m^2^.

A total of 6336 DKD hospitalized patients were included in our current study from October 1st, 2017 to March 30th, 2020. Of these, 42.7% were admitted for poor glycemic control, 26.7% for exacerbation of DKD-related symptoms, 21.4% for cardiovascular disease and 9.2% for diabetes complications. Mean age was 58.0 ± 12.5 years and 36.8% were female. Figure 1 showed the proportion of overall patients classified according to GFR and ACR, with G1-G5 accounting for 36.7%, 21.8%, 10.8%, 12.7%, 14.3% and 3.7% respectively, and A1–A3 accounting for 16.7%, 30.1% and 53.2% respectively.

### Time trends in SGLT2 inhibitors use

At baseline, 781 (12.3%) patients were treated with SGLT2i, with 68.8% of them treated with dapagliflozin, 17.9% with empagliflozin and 13.3% with canagliflozin. The mean age was 53.7 ± 12.7 years, and the majority of the patients were male (75.4%).

**Figure 2:**
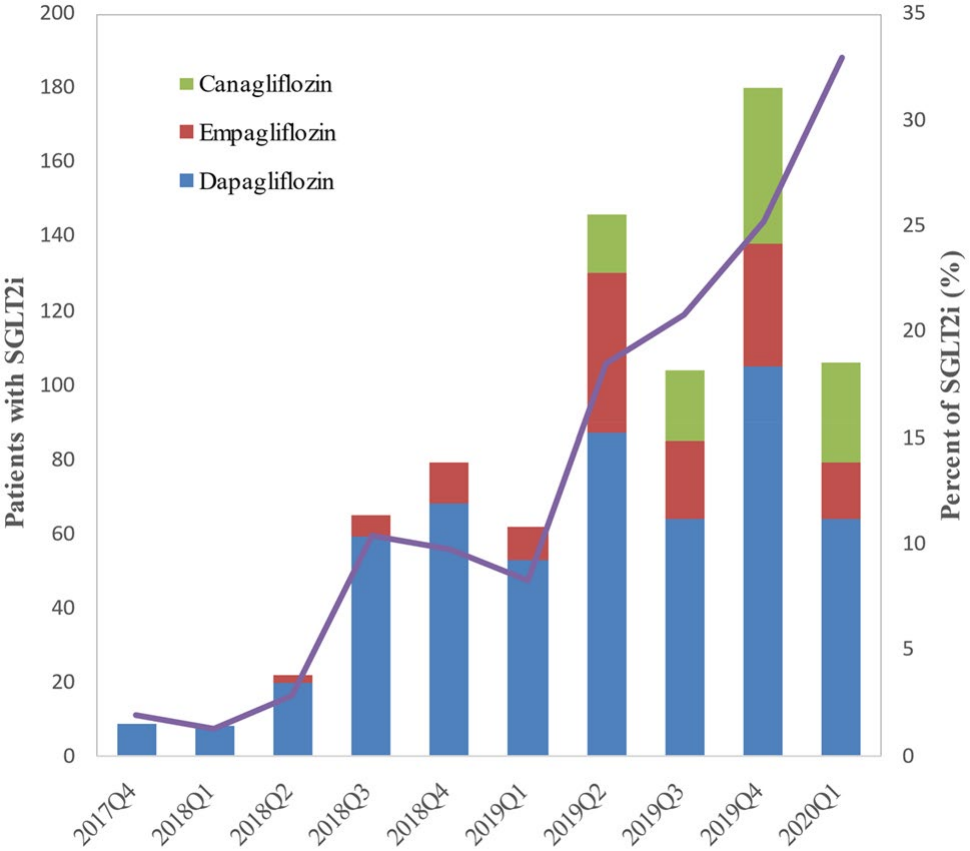
Time trends in proportion using SGLT2i and individual SGLT2i in study population. The stacked bar graphs represent the number of patients using dapagliflozin, empagliflozin and canagliflozin per quarter and the broken line represents the change trend of the overall relative DKD patients using SGLT2i over time.

Figure 2 showed the trend in SGLT2i over time. SGLT2i increased over time, from 1.9% in October 2017 to 33% in March 2020 (*P*_trend_ < 0.001). Meanwhile, the specific agent of SGLT2i also varied in different time segments. Dapagliflozin gradually decreased over time from an initial percentage of 100% to 60.4%. The use of empagliflozin in DKD patients began after March 2018, with slight increase (from 9.1% to 14.2%), while canagliflozin initiated after March 2019 and trended linearly upwards (from 11% to 25.5%).

**Figure 3:**
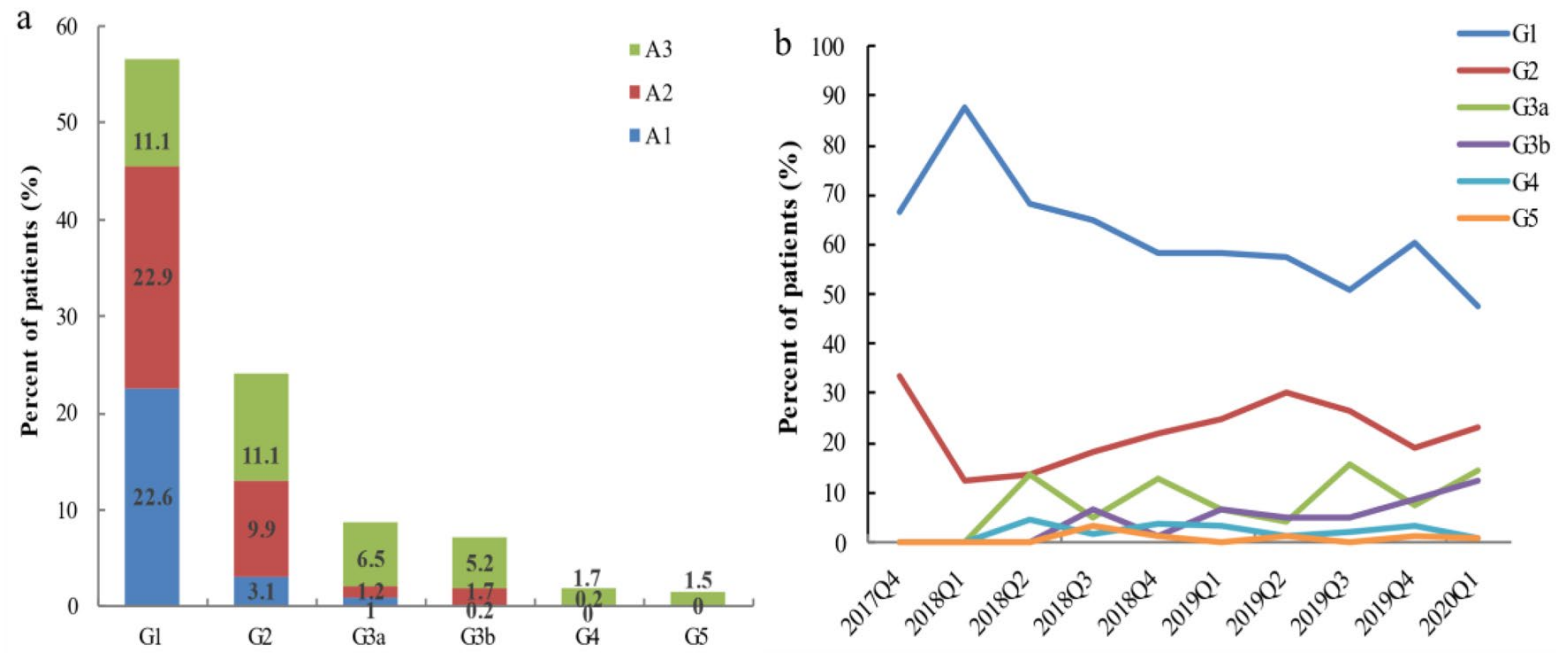
Distribution of GFR and albuminuria in SGLT2i. (**a**) Proportion of patients using SGLT2i in each eGFR and albuminuria category; (**b**) Trends in eGFR status over time in patients using SGLT2i. A1–A3 denote albuminuria category. Albuminuria categories: A1, < 30 mg/g; A2, 30–300 mg/g; A3, > 300 mg/g. G1–G5 denote GFR categories: G1, ≥ 90 ml/min/1.73 m^2^; G2, 60–89 ml/min/1.73 m^2^; G3a, 45–59 ml/min/1.73 m^2^; G3b, 30–44 ml/min/1.73 m^2^; G4, 15–29 ml/min/1.73 m^2^; G5, < 15 ml/min/1.73 m^2^.

As can be seen in Fig. 3a, most patients on SGLT2i were in the G1 and G2 stages (80.7%). Patients in stages A1–A3 accounted for 26.9%, 35.9% and 37.1% respectively. In addition, the temporal trend of eGFR shows that the proportion of patients with CKD3a and CKD3b stages is increasing, from 0% to 14.6% and 12.6% respectively (Fig. 3b).

### Characteristics of patients using SGLT2 inhibitors

As shown in Table 1, patients who used SGLT2 inhibitors performed younger age, higher BMI and HbA1c, shorter duration of diabetes and preserved kidney function, with the higher prevalence of dyslipidemia, cardiovascular disease, diabetic retinopathy and lower prevalence of hypertension, anemia and hyperuricemia. In addition, patients on SGLT2 inhibitor were more likely to be concomitant with ACEI/ARBs, statins, and other hypoglycemic drugs.

**Table 1:** Demographic and clinical characteristics for all patients.

Variables	SGLT2i users (n = 781)	Non-SGLT2i users (n = 5555)	*P* value
Age—yr	53.7 ± 12.7	58.6 ± 12.3	< 0.001
Female sex—no. (%)	192 (24.6)	2135 (38.4)	< 0.001
BMI—kg/m^2^	27.3 ± 4.2	25.6 ± 3.9	< 0.001
Systolic pressure—mmHg	138.9 ± 18.0	141.9 ± 34.6	< 0.001
Diastolic pressure—mmHg	84.6 ± 11.7	83.3 ± 12.4	0.006
Duration of diabetes—yr	9.8 ± 7.4	11.4 ± 7.8	< 0.001
Triglyceride—mmol/l	2.6 (1.8, 3.3)	1.6 (1.1, 2.3)	< 0.001*
LDL-C—mmol/l	2.0 (1.3, 3.1)	2.6 (1.9, 3.4)	< 0.001*
HbA1c—%	9.2 ± 2.0	8.4 ± 2.1	< 0.001
eGFR—ml/min/1.73 m^2^	87.9 ± 28.3	60.4 ± 40.2	< 0.001
Median UACR—mg/g	132.0 (27.0, 649.2)	577.3 (75.7, 3062.1)	< 0.001*
UACR—no. (%)			
< 30 mg/g	129 (26.9)	383 (14.0)	
30–300 mg/g	171 (35.9)	725 (26.5)	
> 300 mg/g	177 (37.1)	1623 (59.4)	
Urea nitrogen—mmol/l	7.0 ± 3.6	11.6 ± 8.6	< 0.001
Hypertension—no. (%)	526 (67.3)	4103 (73.9)	< 0.001
Cardiovascular disease—no. (%)	467 (59.8)	2707 (48.7)	< 0.001
Diabetic retinopathy—no. (%)	264 (33.8)	1397 (25.1)	< 0.001
Anemia—no. (%)	137 (18.4)	2459 (45.0)	< 0.001
Dyslipidemia—no. (%)	691 (92.1)	3707 (71.3)	< 0.001
Hyperuricemia—no. (%)	118 (15.7)	1239 (25.4)	< 0.001
Medication—no. (%)			
ACEI/ARBs	514 (65.8)	2398 (43.2)	< 0.001
Statins	596 (76.3)	3165 (57.0)	< 0.001
Insulin	692 (88.6)	4228 (76.1)	< 0.001
Metformin	576 (73.8)	2052 (36.9)	< 0.001

Data were shown as mean ± standard deviation, median (interquartile) or frequency (percentage). The t-test and chi-squared test were used unless stated otherwise (*Mann–Whitney U-test).

SGLT2i, sodium–glucose cotransporter 2 inhibitor; BMI, body mass index; LDL-C, low density lipoprotein cholesterol; HbA1c, glycosylated hemoglobin; eGFR, estimated glomerular filtration rate, UACR, urine albumin creatine ratio; ACEI/ARBs, angiotensin converting enzyme inhibitors/angiotensin receptor blockers.

### Treatment scheme analysis

**Figure 4:**
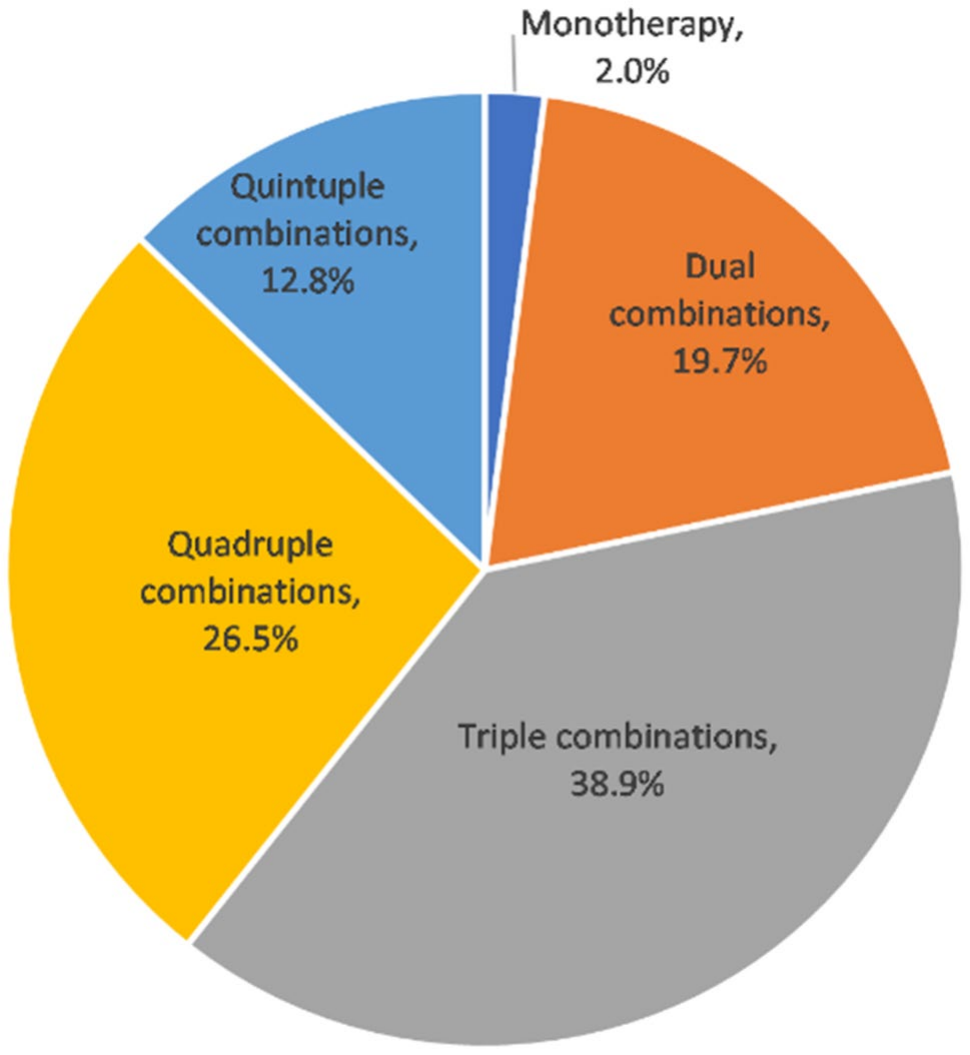
Types of combination with SGLT2i.

**Figure 5:**
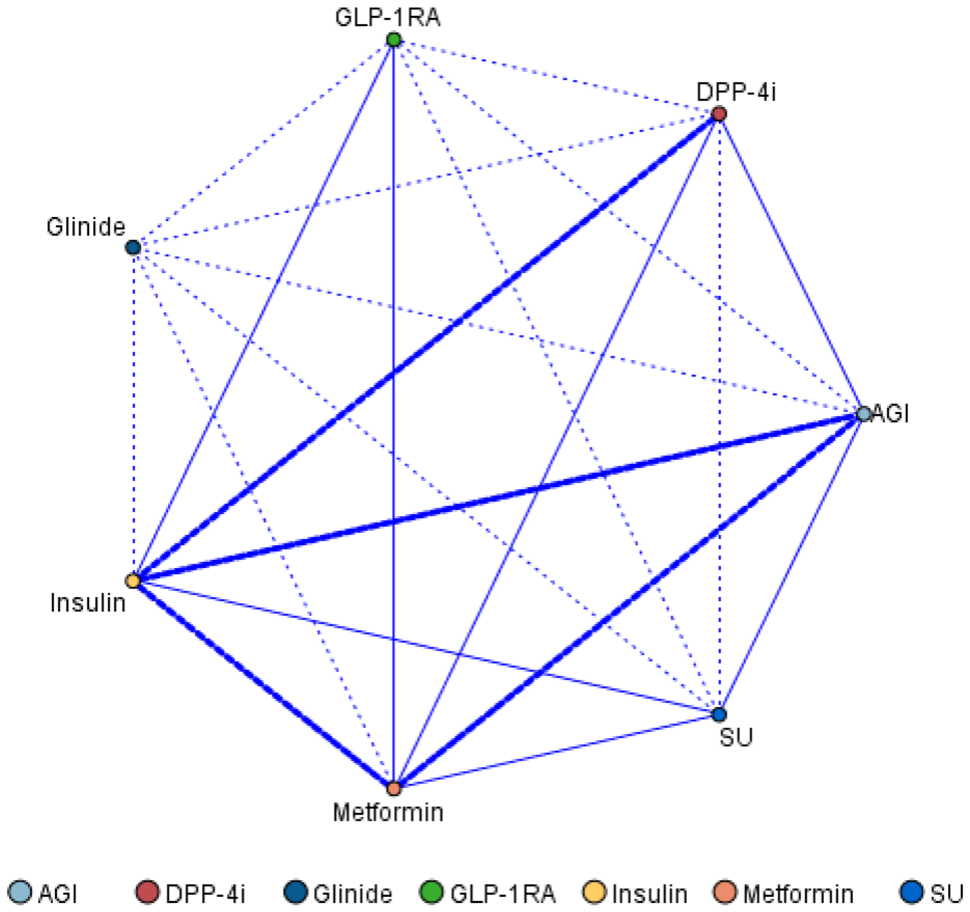
Network diagram of hypoglycemic drugs used in combination with SGLT2i. The network diagram indicates the class and frequency of other hypoglycemic agents used in combination with SGLT2i. Dot refers to each hypoglycemic agent. Line refers to the combination of each two hypoglycemic agents. The combined frequencies ≥ 25% are indicated by a thick line, the combined frequencies ≤ 10% are indicated by a dashed line, and the combined frequencies in between are indicated by a thin solid line. AGI, alpha-glucosidase inhibitors; DPP-4i, dipeptidyl peptidase-4 inhibitors; GLP-1RA, glucagon-like peptide-1 receptor agonists; SU, sulfonylurea.

**Figure 6:**
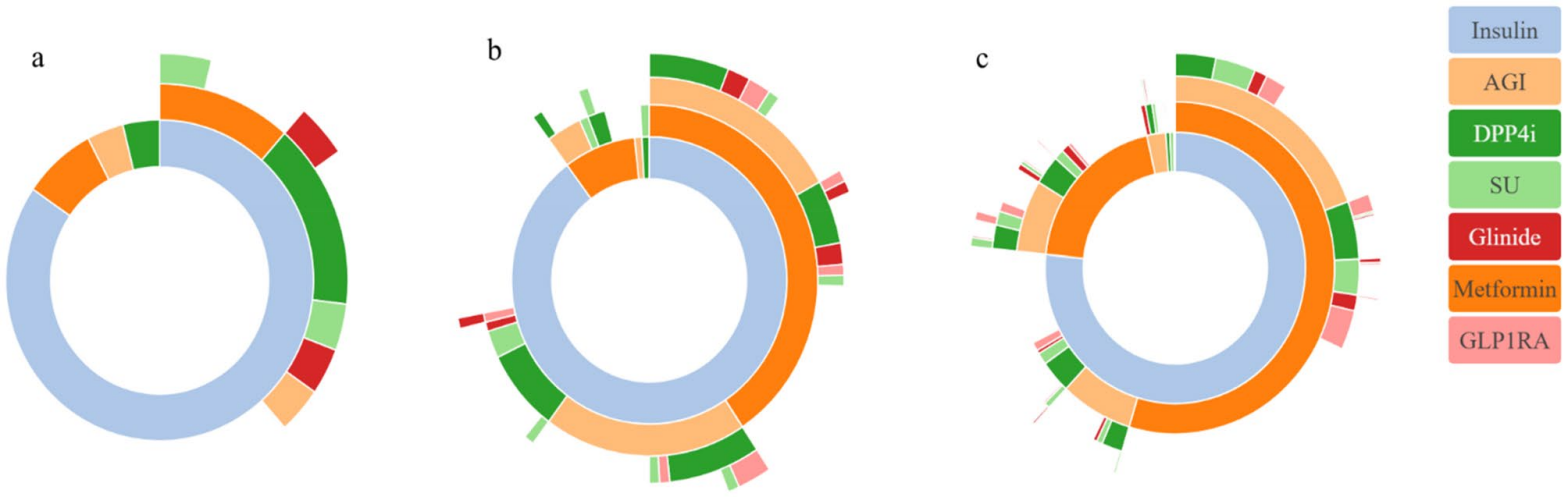
Combination patterns of hypoglycemic drugs in patients with different renal function. (**a**) patients with eGFR < 30 ml/min/1.73 m^2^; (**b**) patients with eGFR 30–59 ml/min/1.73 m^2^; (**c**) patients with eGFR ≥ 60 ml/min/1.73 m^2^. The inner circle refers to the proportion of patients using SGLT2 inhibitor with other ONE hypoglycemic drug, such as combination of SGLT2i and insulin (the light blue part). Each part of outer circle refers to the proportion of patients using SGLT2 inhibitor with other TWO, THREE, FOUR hypoglycemic drugs, respectively. AGI, alpha-glucosidase inhibitors; DPP4i, dipeptidyl peptidase-4 inhibitors; GLP1RA, glucagon-like peptide-1 receptor agonists; SU, sulfonylurea.

Among 781 DKD patients treated with SGLT2i, 765 (98.0%) were combined with other hypoglycemic drugs. Of them, patients on triple and quadruple combinations were more numerous, accounting for 38.9% and 26.5%, respectively (Fig. 4). The network diagram showed that SGLT2i was most commonly combined with insulin, metformin, AGI, and DPP-4i (Fig. 5). The association rules showed that the top five modes of combination with SGLT2i in terms of support were insulin, metformin, insulin + metformin, AGI, and insulin + AGI (Table 2). There were 613 patients with eGFR ≥ 60 ml/min/1.73 m^2^, 119 patients with eGFR 30–59 ml/min/1.73 m^2^, and 26 patients with eGFR < 30 ml/min/1.73 m^2^. The sunburst chart displayed the combination patterns in patients with different renal function, with the number of combinations reducing as eGFR declines (Fig. 6). Association rules showed that the combination patterns of patients with eGFR ≥ 60 ml/min/1.73 m^2^ and eGFR 30–59 ml/min/1.73 m^2^ were consistent with the overall combination patterns, while for patients with eGFR < 30 ml/min/1.73 m^2^, the top five combination modes according to the support were insulin, metformin, DPP-4i, insulin + DPP-4i, and insulin + metformin (Table 2).

**Table 2:** Apriori algorithm of association rules.

Group	Model	Support (%)	Confidence (%)
Total	Insulin	79.54	100.00
	Metformin	64.71	100.00
	Insulin + metformin	47.57	100.00
	AGI	35.17	100.00
	Insulin + AGI	28.26	100.00
eGFR- ml/min/1.73 m^2^			
≥ 60	Insulin	78.00	100.00
	Metformin	69.54	100.00
	Insulin + metformin	51.10	100.00
	AGI	36.21	100.00
	Insulin + AGI	27.07	100.00
30–59	Insulin	89.08	100.00
	Metformin	48.74	100.00
	Insulin + metformin	40.34	100.00
	AGI	40.34	100.00
	Insulin + AGI	36.13	100.00
< 30	Insulin	81.48	100.00
	Metformin	18.52	100.00
	DPP-4i	18.52	100.00
	Insulin + DPP-4i	14.81	100.00
	Insulin + metformin	11.11	100.00

eGFR, estimated glomerular filtration rate; AGI, alpha-glucosidase inhibitors; DPP-4i, dipeptidyl peptidase-4 inhibitors.

### Factors influencing the use of SGLT2 inhibitors

**Figure 7:**
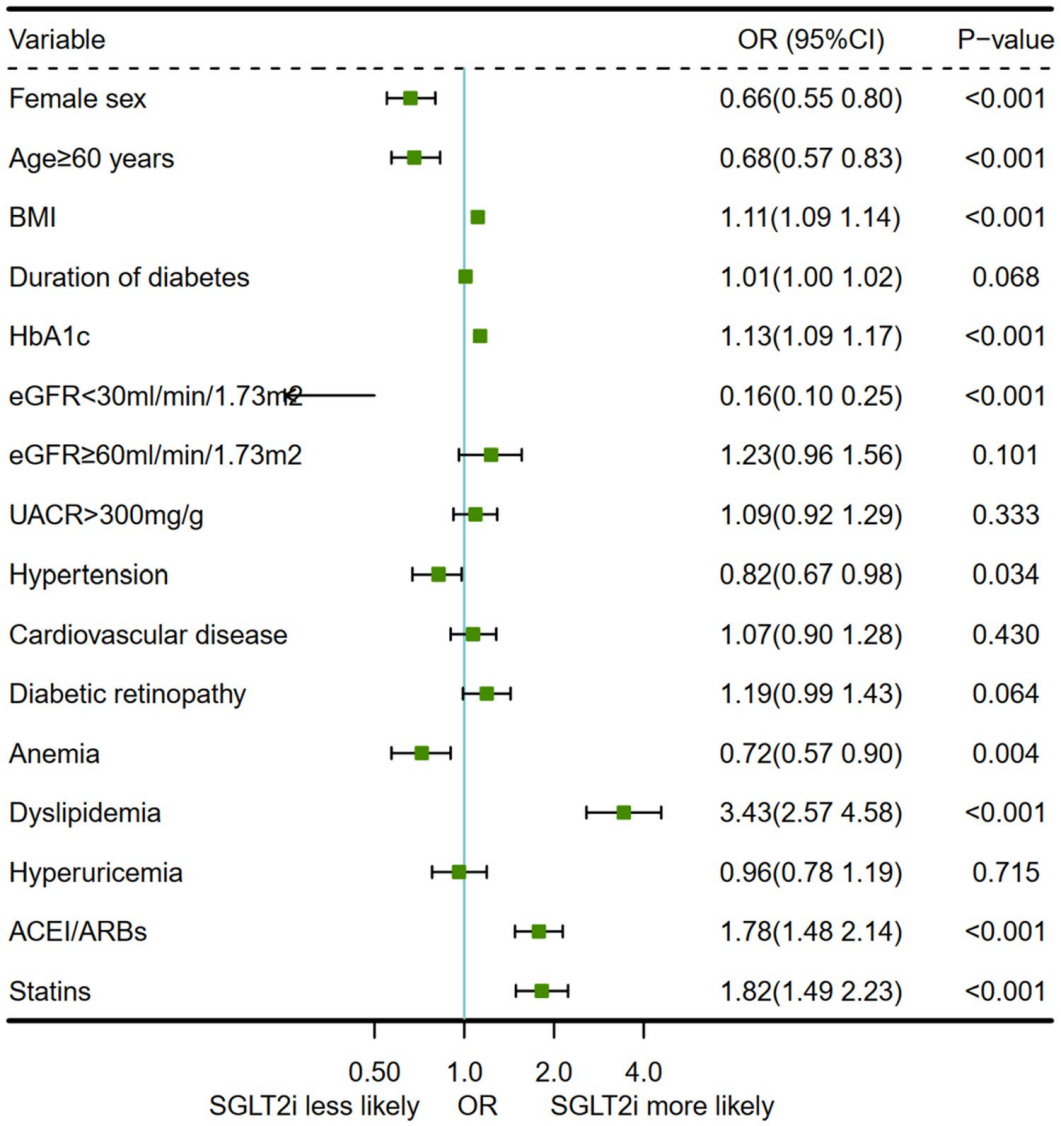
Factors influencing the use of SGLT2 inhibitor. SGLT2i, sodium–glucose cotransporter 2 inhibitor; BMI, body mass index; HbA1c, glycosylated hemoglobin; eGFR, estimated glomerular filtration rate; UACR, urine albumin creatine ratio; CVD, cardiovascular disease; ACEI/ARBs, angiotensin converting enzyme inhibitors/angiotensin receptor blockers.

There was no assumption of multicollinearity between variables. Results of multivariate logistic regression indicated that higher BMI, higher HbA1c level and having dyslipidemia increased the odds of using SGLT2 inhibitor, with an OR of 1.11 (95% CI 1.09–1.14), 1.13 (95% CI 1.09–1.17) and 3.43 (95% CI 2.57–4.58), respectively. Patients on SGLT2 inhibitor more treated with ACEI/ARBs (OR: 1.78, 95% CI 1.48–2.14) and statins (OR: 1.82, 95% CI 1.49–2.23). On the contrary, patients who aged ≥ 60 years (OR: 0.68, 95% CI 0.57–0.83), having history of hypertension (OR: 0.82, 95% CI 0.67–0.98) and anemia (OR: 0.72, 95% CI 0.57–0.90) were less likely to use SGLT2 inhibitor; moreover, patients with eGFR < 30 ml/min/1.73 m^2^ were less likely to use an SGLT2i than eGFR between 30 and 59 ml/min/1.73 m^2^ (OR: 0.16, 95% CI 0.10–0.25) (Fig. 7).

### Sensitivity analysis

There were 3812 DKD patients with eGFR ≥ 45 ml/min/1.73 m^2^, of whom 682 patients (17.9%) were treated with SGLT2i. Logistic regression analyses revealed that SGLT2 inhibitor initiation had correlation with younger age, higher BMI, higher HbA1c, better kidney function, dyslipidemia, no history of hypertension and use of ACEI/ARBs and statins (Supplementary Fig. S1).

## Discussion

In this study, we analyzed the real-world data of SGLT2 inhibitors using in DKD patients. In contrast to previous studies, this study analyzed various aspects of the population characteristics, combination use and factors influencing the use of SGLT2i in patients with DKD. The results show that the proportion of SGLT2i using increased significantly since 2017, but there were still only 12.3% of DKD patients received it. SGLT2i was initiated most often for patients with younger than 60 years, higher BMI, higher HbA1c, preserved kidney function, and combined using ACEI/ARB and statins. Moreover, 98% of DKD patients treated with SGLT2i were combined with other hypoglycemic agents, with insulin, metformin, and AGI being the main combination agents.

The increasing use of SGLT2 inhibitors in DKD patients was associated with the continuous updating of RCTs results. In many countries, including China, SGLT2 inhibitors were not recommended for patients with DKD and eGFR < 45 mL/min/1.73 m^2^ because of their reduced glycemic efficacy^14–16^. However, results of CREDENCE and DAPA-CKD trial indicated that SGLT2 inhibitors could reduce the risk of renal events with no restrictions of baseline eGFR and thus were recommended for T2DM patients with DKD and eGFR ≥ 30 ml/min/1.73 m^2^^8–10,17^. Clinical trial outcome data may be the driver of drug selection^18^. Dapagliflozin was the first commercially approved SGLT2i for the treatment of T2DM. It was launched in China in March 2017 and is the most prescribed drug in our study. With publication of the EMPA-REG outcome trial, empagliflozin increased cardiovascular indications, canagliflozin received an FDA black box warning for a potential risk of lower limb amputation, and the prescribing rate of empagliflozin increased, the trend also seen in the drug market^7,12,19^. However, in our current, the number of patients who used empagliflozin was not increased significantly, which suggested the subsequent changes in prescription patterns need to be further observed.

Our study found that, although the proportion of SGLT2i increased year by year, the overall utilization rate of SGLT2i was low in DKD patients. Previous studies have also found low rates of SGLT2i use, partly due to the high out-of-pocket costs associated with SGLT2i as a new drug^20,21^. In China, on the other hand, SGLT2i were about four times less expensive than before, following their inclusion in health insurance in November 2019. Therefore, the cost of the drug may not be the reason for the low application of SGLT2 inhibitors. This phenomenon partly attributed to the clinical inertia of physicians who were not fully aware of the serious cardiovascular risk in DKD patients and the cardiorenal benefits of cardiovascular outcome trials^22,23^. Also, patients with long-term duration of diabetes are more susceptible to be influenced by clinical inertia. They feel more comfortable when using regular or “old” therapy plan rather than trying new treatment while their physicians are also reluctant to change medications when blood glucose control is in good range, regardless of the cardiorenal risk^23^.

We found that patients on SGLT2i had high baseline albuminuria levels, accounting for 73% of patients in stages A2 and A3. Guidelines also recommend the use of SGLT2i in T2DM patients with CKD, especially those with UACR > 300 mg/g^24^. SGLT2i can perform reno-protective effects by reducing albuminuria, mainly because of reducing intraglomerular pressure and glomerular hyperfiltration.

Tumminia et al. find that SGLT2i treatment was associated with significant improvements in HbA1c and BMI levels in patients aged ≥ 65 years and with similar renal safety and tolerability regardless of patients age^25^. However, in our study we found that relatively few patients aged ≥ 60 years were using SGLT2i, possibly because the elderly are more prone to various complications and side effects of the use of new drugs, rational individual administration should be made^26^. Those who use SGLT2i had higher BMI, study had shown that obesity drives treatment choices more than cardiorenal risk when comparing with GLP-1RA, and DPP4i^27^. Patients with T2DM who were treated with SGLT2i lose 1 to 3 kg of body weight, mainly as a result of energy and water loss due to osmotic diuresis^28^. Weight loss of 5–10% in T2DM patients is related to the beneficial effects on blood glucose, blood pressure and triglycerides^29^. Our findings indicated that patients treated with SGLT2i were more likely to be in the early stages of CKD with poor glycemic control. It should not be limited to hypoglycemic centered, studies had shown that intensive glycemic control did not reduce the risk of advanced CKD progression or cardiovascular death^30^. Cardiovascular and renal risk should be considered independently of HbA1c, and ADA standard (2022) places more emphasis on the consideration of cardiac and renal complications in drug selection^31–33^. Guideline recommendations and real-world studies showed renal benefits of SGLT2i regardless of baseline eGFR and albuminuria status, and may extend the scope of eGFR use for SGLT2i^34^.

Patients using SGLT2i are mostly on combination with ACEI/ARB, which may have a synergistic effect on the kidney^35^. Several studies had shown that the combination of SGLT2i and ACEI/ARB had a better protective effect on heart and kidney, achieved better control of blood glucose, blood pressure and body weight^36–38^.

Our study found that SGLT2 inhibitors were mostly used in combination with conventional hypoglycemic agents, and the most patients combined with insulin, followed by metformin. The risk of hypoglycemia may increase when T2DM patients with CKD are treated with multiple hypoglycemic drugs, including insulin, sulfonylureas, etc. Therefore, the dose of insulin should be reduced appropriately when combined with insulin. Dario et al. showed basal insulin plus dapagliflozin had a similar effect on glycemic control as basal-bolus insulin regimen, with a reduction in total insulin dose and a lower risk of hypoglycemia^39^. In several large RCT studies, the combination of metformin and SGLT2i was used in more than 50% of the patients, the results of these studies also demonstrated significant benefits in attenuating renal impairment, preventing ESRD and reducing mortality from renal disease^6–8^. Recently, the joint consensus from ADA and KIDIGO recommended early initiation of metformin in combination with SGLT2i in patients with T2DM and CKD^40^. GLP-1RA is a hypoglycemic drug with renal benefit and studies had shown to have synergistic effects with SGLT2i in terms of glycemic control and reduced risk of cardiovascular and renal events^41,42^. However, only 9.8% of the patients in our study were combined with GLP-1RA. In addition, we found that 12.8% of patients used more than 4 types of hypoglycemic drugs. With too many types of medication, patients are less likely to comply, and are more likely to take the wrong medication, miss a dose or take too many doses, while increasing the cost burden on patients. Clinicians should develop a reasonable hypoglycemic regimen according to the individual situation of patients and aim to achieve the desired effect with the least type and dose of drugs.

This study has a large sample size, with patients from different provinces in China. However, the study is a single-center study and is only indicative of drug use in central China; Further research is needed to refine the situation in other regions.

In a real-world population of DKD, use of SGLT2i is low and most of the patients used were younger and in the early stages of chronic kidney disease with poor glycemic control. Therefore, under the conditions for the use of SGLT2i, the scope of use should be moderately expanded to overcome therapeutic inertia. In addition, individualized treatment for patients, as far as possible to play the role of cardiovascular and kidney protection.

## Supplementary Information

Supplementary Figure S1.

## Data Availability

The dataset analyzed during the current study is available from the corresponding author on reasonable request.
