# Supplementary material for: Integrated analysis for treatment scheme of sodium–glucose cotransporter 2 inhibitors in patients with diabetic kidney disease: a real-world study
Source: Sci Rep. 2023 Apr 12;13:5969. doi: 10.1038/s41598-023-33211-1 (PMC10097684; doi:10.1038/s41598-023-33211-1)
Supplement: Supplementary file 1 — Supplementary Figure S1. [file 41598_2023_33211_MOESM1_ESM.pdf]

Integrated analysis for treatment scheme of sodium-glucose cotransporter 2 inhibitors in patients with diabetic kidney disease: a real-world study

Li Fang[1,2,3,4,5], Guangpu Li[1,2,3,4,5], Jingjing Ren[1,2,3,4,5], Jiayu Duan[1,2,3,4,5*], Jiancheng Dong[5*] and Zhangsuo Liu[1,2,3,4*]

[1]Department of Integrated Traditional and Western Nephrology, the First Affiliated Hospital of Zhengzhou University, Zhengzhou, China
[2]Research Institute of Nephrology, Zhengzhou University, Zhengzhou, China
[3]Henan Province Research Center For Kidney Disease, Zhengzhou, China
[4]Key Laboratory of Precision Diagnosis and Treatment for Chronic Kidney Disease in Henan Province, Zhengzhou, China
[5]Clinical Research Center of Big-data, the First Affiliated Hospital of Zhengzhou University, Zhengzhou, China
*Correspondence to: Jiayu Duan, jyduan@hotmail.com
Jiancheng Dong, fccjcdong@zzu.edu.cn
Zhangsuo Liu, zhangsuoliu@zzu.edu.cn

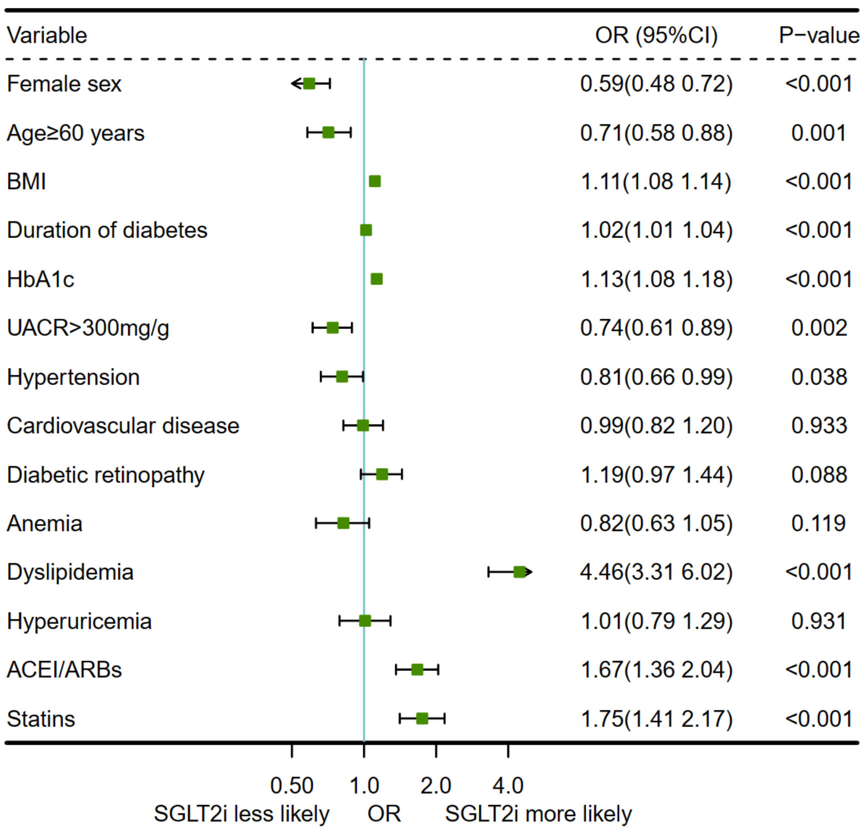

Supplementary Fig S1. Subgroup analyses in eGFR ≥ 45 ml/min/1.73m^2 patients.

Abbreviations: SGLT2i, sodium-glucose cotransporter 2 inhibitor; BMI, body mass index; HbA1c, glycosylated hemoglobin; UACR, urine albumin creatine ratio; CVD, cardiovascular disease; ACEI/ARBs, angiotensin converting enzyme inhibitors/angiotensin receptor blockers; eGFR, estimated glomerular filtration rate.
